# Implication of Potassium Channels in the Pathophysiology of Pulmonary Arterial Hypertension

**DOI:** 10.3390/biom10091261

**Published:** 2020-09-01

**Authors:** Hélène Le Ribeuz, Véronique Capuano, Barbara Girerd, Marc Humbert, David Montani, Fabrice Antigny

**Affiliations:** 1 Faculté de Médecine, Université Paris-Saclay, 94270 Le Kremlin-Bicêtre, France; helene.leribeuz@aol.com (H.L.R.); veronique.capuano@universite-paris-saclay.fr (V.C.); barbara.girerd19@gmail.com (B.G.); mjc.humbert@gmail.com (M.H.); david.montani@aphp.fr (D.M.); 2 INSERM UMR_S 999, Hypertension pulmonaire, Physiopathologie et Innovation Thérapeutique, Hôpital Marie Lannelongue, 92350 Le Plessis-Robinson, France; 3 Assistance Publique—Hôpitaux de Paris (AP-HP), Service de Pneumologie et Soins Intensifs Respiratoires, Centre de Référence de l’Hypertension Pulmonaire, Hôpital Bicêtre, 94270 Le Kremlin-Bicêtre, France

**Keywords:** KCNK3, ABCC8, KCNA5, ABCC9, K2P3.1, SUR1, Kv1.5, SUR2

## Abstract

Pulmonary arterial hypertension (PAH) is a rare and severe cardiopulmonary disease without curative treatments. PAH is a multifactorial disease that involves genetic predisposition, epigenetic factors, and environmental factors (drugs, toxins, viruses, hypoxia, and inflammation), which contribute to the initiation or development of irreversible remodeling of the pulmonary vessels. The recent identification of loss-of-function mutations in *KCNK3* (KCNK3 or TASK-1) and *ABCC8* (SUR1), or gain-of-function mutations in *ABCC9* (SUR2), as well as polymorphisms in *KCNA5* (Kv1.5), which encode two potassium (K^+^) channels and two K^+^ channel regulatory subunits, has revived the interest of ion channels in PAH. This review focuses on KCNK3, SUR1, SUR2, and Kv1.5 channels in pulmonary vasculature and discusses their pathophysiological contribution to and therapeutic potential in PAH.

## 1. Introduction

Pulmonary arterial hypertension (PAH) is a rare and severe cardiopulmonary disease. PAH is a consequence of the progressive obstruction of distal pulmonary arteries (<500 µm in diameter). These modifications of the pulmonary vascular bed increase the right ventricular (RV) afterload, leading to RV hypertrophy and then RV failure and death [1]. PAH is defined by an elevation of the mean pulmonary arterial pressure (mPAP) > 25 mmHg, a pulmonary artery wedge pressure ≤15 mmHg, and a pulmonary vascular resistance (PVR) ≥ 3 woods units [2]. PAH is still incurable, and lung transplantation is the only therapeutic option [3]. PAH may be idiopathic (iPAH, when the causes remain unknown), heritable (hPAH), induced by drugs, induced by toxins, or associated with other pathologies (connective tissue disease, HIV infection, portal hypertension, congenital heart disease, and schistosomiasis) [4].

The pathophysiology of PAH is complex and multifactorial. PAH is mostly caused by a dysfunction of pulmonary arterial endothelial cells (PAECs) and pulmonary arterial smooth muscle cells (PASMCs) characterized by abnormal pulmonary artery vasoconstriction and an unbalance between proliferation and apoptosis in favor of cell proliferation and apoptosis resistance [5]. PAEC dysfunction is characterized by an exaggerated production of vasoconstrictive and mitogen molecules, including endothelin-1 (ET-1), serotonin (5-HT), or thromboxane A2 (TXA) [6,7].

Most hPAH cases are due to a pathogenic mutation in the *bone morphogenic protein receptor type II* gene *(BMPR2)* (approximately 85%) [8]. However, 17 other PAH-predisposing genes have recently been identified, including *EIF2AK4, ACVRL1, TBX4, GDF2, SOX17, ENG, KCNK3, ABCC8, ATP13A3, SMAD9, AQP1, CAV1, BMP10, SMAD4, SMAD1, G6PD*, and *KDR* [9,10,11]. Among all PAH-predisposing genes, two of them encode potassium (K^+^) channels or regulatory subunits of K^+^ channels, namely *potassium channel subfamily K member 3* (*KCNK3*) and *ATP-binding cassette subfamily C member 8* (*ABCC8*), respectively. *KCNK3* and *ABCC8* represent the only two channelopathies described in PAH. K^+^ channels are transmembrane proteins that are responsible for the circulation of K^+^ ions according to the concentration gradient of K^+^ from the cytoplasm ([K^+^] = 140 mM) towards the extracellular medium ([K^+^] = 5 mM). K^+^ channels are categorized into three major groups based on their pharmacological and electrophysiological properties and structures: Inward-rectifying potassium channels (Kir), including ATP-sensitive potassium channels (KATP); voltage-gated potassium channels (Kv); and two pore domain K^+^ channels (K2P). *ABCC8* and *ABCC9* encode two different regulatory subunits of the Kir channel (KATP channels), *KCNA5* encodes a Kv channel, and *KCNK3* encodes a K2P channel. K^+^ channels are the most important regulator of the resting membrane potential (Em). The inhibition, downregulation, or loss-of-function (LOF) mutation in K^+^ channels leads to plasma membrane depolarization of excitable and non-excitable cells. In excitable cells, Em depolarization activates voltage-gated Ca^2+^ channels, leading to an increase in the intracellular Ca^2+^ concentration [Ca^2+^]_i_ [12,13]. This increase of [Ca^2+^]_i_ enhances PASMC constriction and proliferation [14], while the change in the intracellular K^+^ concentration may modify the cell volume and apoptotic status of the cells [15]. This review focuses on PAH-predisposing genes that encode K^+^ channels, their physiological functions in pulmonary vasculature and RV, their contributions to the pathophysiology of PAH, and their therapeutic potential in PAH.

### 1.1. KCNK3

**Figure 1:**
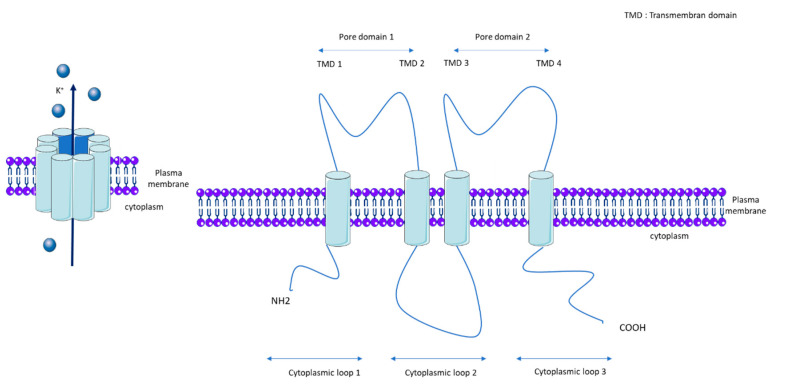
General molecular architecture of two pore potassium channels (K2P). TMD: Transmembrane domain.

KCNK3, also called TASK-1 (TWIK-related acid sensitive K^+^) or K2P3.1, is a member of the K2P channel family. K2Ps are background K^+^ conductance channels that contribute to resting membrane potentials. The K2P channels are composed of two subunits, each with two pore domains that together form a single pore highly selective for K^+^ (Figure 1). The K2P family is comprised of 15 members and the following five subfamilies, which are classified according to their pharmacological and electrophysiological properties: TWIK, Tandem of P domains in Weak Inward rectifier K^+^ channel; TREK/TRAAK, TWIK-Related K^+^ channel/TWIK-Related Arachidonic Acid-Stimulated K^+^ channel; THIK, Tandem pore domain Halothane-Inhibited K^+^ channel; TALK, TWIK-related Alkaline pH-Activated K^+^ channel; and TASK, TWIK-related Acid-Sensitive K^+^ channel [13]. Gardener et al. demonstrated the expression of mRNA of TASK-1, TASK-2, THIK-1, TRAAK, and TREK-1 in rat pulmonary arteries, and confirmed the protein expression of TASK-1, TASK-2, TREK-1, and TWIK-2 [16]. *kcnk6*-deficient mice develop spontaneous pulmonary hypertension (PH) at between 8 and 20 weeks [17]. Nonetheless, we recently found that *KCNK6* mRNA expression is unchanged in the pulmonary artery of iPAH patients [18].

### 1.2. KCNK3 Properties and Regulatory Mechanisms

KCNK3 can exist in a homodimer or heterodimer conformation, coupling with KCNK9 (TASK-3) [19]. However, KCNK9 (TASK-3) is not expressed in the lung [20]. The KCNK3 channel is inhibited by a wide range of molecules, including 4-aminopyridine. Contrary to other K^+^ channels, the KCNK3 channel is insensitive to tetraethylammonium (TEA), glibenclamide, cesium, and intracellular [Ca^2+^]_i_.

KCNK3 is specifically blocked by a number of compounds, such as the aromatic carbonamide A293 developed by Sanofi. As demonstrated by Putzke et al., A293 at a 1 μM concentration inhibited 75% of hKCNK3 and 50% of hKCNK9 currents, while other K^+^ channels tested (TASK2, TASK4, TREK-1, Kv1.1, Kv1.3, Kv1.4, Kv1.5, Kv4.3, and hERG) were inhibited by less than 12% [21].

Another KCNK3 inhibitor, the A1899 compound, as shown by Streit et al., when administered at 100 nM, inhibited 90% of hKCNK3 and 20% of KCNK9, while the functions of other K^+^ channels tested (TASK-2, TASK-4, TREK-1, Kv1.1, Kv1.3, Kv1.5, Kv3.1, Kv4.3, HCN1, HCN2, Kir1.1, and Kir2.1) were inhibited by less than 11% [22]. The commercial ML365 compound displays an IC50 = 16 nM for hKCNK3, with more than a 60-fold selectivity for hKCNK3 over the hKCNK9 channel [23,24].

The local anesthetics lidocaine and bupivacaine non-specifically inhibit KCNK3 at relatively high concentrations [25]. In addition, anandamide and methanandamide, doxapram, and PKTHPP are nonselective blockers of KCNK3 and KCNK9 [26,27,28,29].

To demonstrate the involvement of KCNK3 dysfunction in PAH in vivo, we chronically exposed control rats to the A293 compound (2 mg/kg/d) and found that A293-exposed rats developed significant early signs of PH. Importantly, KCNK9 is not expressed in lungs from rats [20,30] and thus cannot be part of the K^+^ current. We were able to isolate KCNK3 from other outward-K^+^ currents in vitro, using the KCNK3 blocker A293 [24,30]. KCNK3-specific inhibition points to a role of KCNK3 in cell proliferation in control human PASMCs [20].

KCNK3 is sensitive to extracellular pH variations. KCNK3 is 100% inhibited by pH 6.4, 50–60% activated at a physiological pH (7.4), and fully activated by pH 8.4 [31]. Local inflammation can induce extracellular pH acidification. The exaggerated lung perivascular inflammation observed in PAH patients [32] could enhance perivascular acidification, partly mediating the loss of the KCNK3 function, and contribute to PAH pathogenesis by promoting pulmonary artery vasoconstriction and PASMC proliferation.

The KCNK3 channel function is reduced by hypoxia stimuli [33]. KCNK3 can mediate the initiation of hypoxia-induced depolarization in PASMCs, contributing to hypoxic pulmonary vasoconstriction.

Endothelial cell dysfunction contributes to PAH pathogenesis by the overproduction of vasoconstrictive and angioproliferative molecules, such as endothelin-1 (ET-1) and serotonin (5-HT) [6,7]. KCNK3 is inhibited by the activation of Gq-protein coupled receptors (GqPCRs), including ET-1. Downstream GqPCR signaling, such as PKC, was suggested to mediate this inhibition. In addition, ET-1 can stimulate Rho-kinase to inhibit the KCNK3 function [34,35,36]. Therefore, the overproduction of ET-1 and 5-HT in PAH partly mediates the loss of the KCNK3 function, which contributes to PAH pathogenesis by promoting pulmonary artery vasoconstriction and PASMC proliferation. The activation of PLC via Gq proteins [37] inhibits KCNK3 via the diacylglycerol (DAG), PKC [38,39]. In addition, the KCNK3 function is enhanced by the protein kinase A (PKA)-dependent phosphorylation of KCNK3 [33], contributing to the vasorelaxing properties of prostanoids. The KCNK3 function is also activated by protein kinase G (PKG) [40], contributing to the vasorelaxating properties of PDE5 inhibitors or guanylate cyclase activators. Indeed, we recently found that *Kcnk3*-LOF-mutated rats are resistant to pulmonary artery relaxation mediated by sildenafil, which is a PDE5 inhibitor [20].

**Figure 2:**
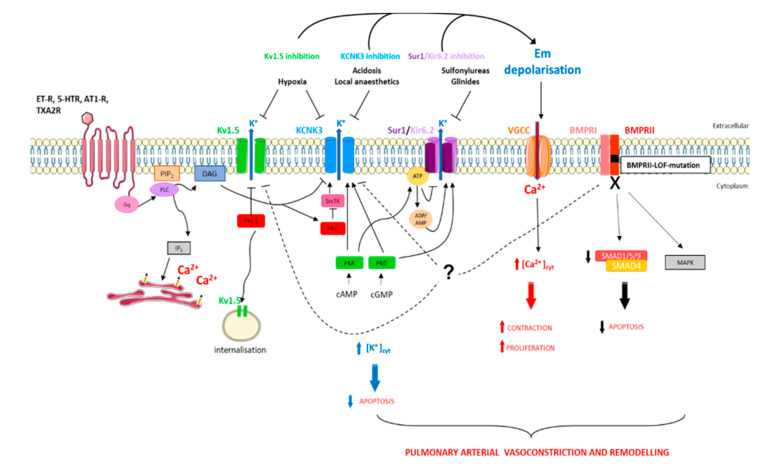
Schematic representation of pathways implicated in K^+^ regulation in pulmonary artery smooth muscle cells in the context of pulmonary arterial hypertension (PAH). [Ca^2+^]_cyt_, cytoplasmic calcium concentration; [K^+^]_cyt_, cytoplasmic potassium concentration; 5-HTR, serotonin receptor; AT1-R, angiotensin 1 receptor; BMPRI, BMP receptor type I; BMPRII, BMP receptor type II; cAMP, cyclic adenosine monophosphate; cGMP, cyclic guanosine monophosphate; DAG, diacylglycerol; Em, membrane resting potential; ET-R, endothelin receptor; IP3, inositol-1,4,5-triphosphate; MAPK, mitogen-activated protein kinases; PIP2, phosphatidylinositol-4,5-biphosphate; PKA, protein kinase A; PKC, protein kinase C; PKG, protein kinase G; PLC, phospholipase C; SMAD, mothers against decapentaplegic homologue; SrcTK, Src family tyrosine kinase; TXA2R, thromboxan A2 receptor.

Moreover the inhibition of Src tyrosine kinase decreases the KCNK3 function [41], which can contribute to the pathogenesis of Dasatinib-(tyrosine kinase inhibitor)-induced PAH, since Dasatinib is known to inhibit Src tyrosine kinase [42]. The regulatory mechanisms of KCNK3 are summarized in Figure 2.

### 1.3. KCNK3 Function and Expression in Pulmonary Vasculature and RV

KCNK3 is expressed in several cell types, including heart cells, lung cells, PASMCs, and PAECs [13,43]. Patch-clamp recording has demonstrated that KCNK3 is functionally expressed in cultured human PASMCs [30,33]. The knockdown of *KCNK3* in human PASMCs or the pharmacological inhibition of KCNK3 in isolated rat PASMCs leads to the depolarization of PASMC Em, demonstrating that KCNK3 contributes to the Em of PASMCs [30,33]. Moreover, plasma membranes of freshly isolated PASMCs from *Kcnk3*-LOF-mutated rats are depolarized compared to PASMCs isolated from WT rats [20]. In association with PASMC depolarization, we found that *Kcnk3* pharmacological inhibition or *Kcnk3*-LOF-mutation causes PA vasoconstriction in rats [19,29]. In contrast to humans and rats, KCNK3 is not functional in mouse PASMCs [44]. 

At the cardiac level, KCNK3 is expressed in atria, ventricular tissues, and heart-conducting tissues in humans, mice, and rats [21,43,45]. Through whole-cell patch-clamp recordings, we found that KCNK3 is functionally expressed in adult rat RV cardiomyocytes, contributing to RV cardiomyocyte excitability (Em and action potential repolarization) [20,46]. In humans, KCNK3 contributes to action potential repolarization and the resting membrane potential of atrial cardiomyocytes. KCNK3 is a key actor of atrial fibrillation [47]. Moreover, the knockdown of KCNK3 in human-induced pluripotent stem cell (iPSC)-derived cardiomyocytes results in a significant prolongation of action potential duration, suggesting that KCNK3 also contributes to action potential repolarization in human ventricular cardiomyocytes [48]. Importantly, patients who develop PH with atrial fibrillation have a higher mortality than PH patients without atrial fibrillation [49], suggesting that *KCNK3*-LOF mutations or KCNK3-dyfunction could also contribute to the clinical deterioration of PH favoring the occurrence of atrial fibrillation.

### 1.4. KCNK3 Mutations in PAH

In 2013, Ma et al. identified six heterozygous mutations in *KCNK3* in families with hPAH and iPAH patients [50]. Four years later, two additional *KCNK3* mutations were identified in a Spanish cohort of PAH patients. One of the mutations (p.Gly106Arg) in a homozygous state is associated with an early and aggressive form of PAH [51]. Best et al. identified two additional mutations [52], and one additional mutation has been identified in Chinese pediatric PAH patients [53]. In 2020, a new mutation in *KCNK3* was identified in a Dutch national cohort of children with PAH [54].

To date, 12 different *KCNK3* mutations have been identified in 19 patients [11,50,51,52,53,54,55]. 

**Figure 3:**
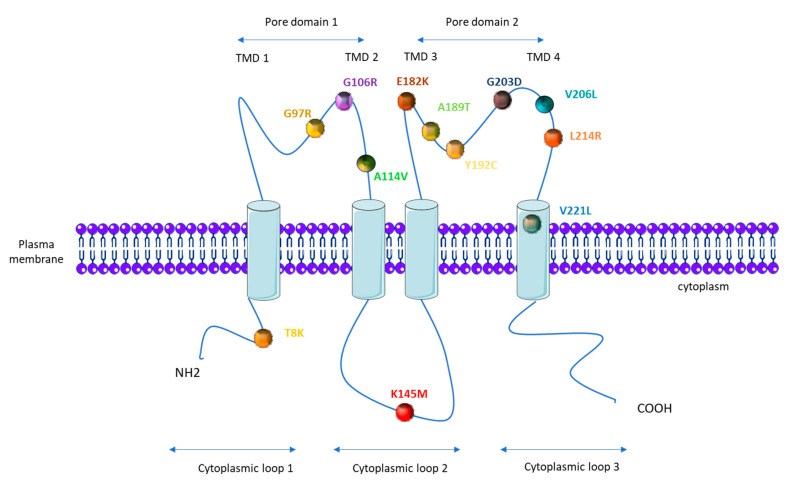
Topological analysis of the human KCNK3/TASK-1 channel. Positions indicate the mutations identified by Ma et al. [50], Navas et al. [51], Zhang [53], and Haarman [54].

PAH patients that carry *KCNK3* mutations are younger at the time of diagnosis (median age of 28 years compared to 42 years for iPAH patients), and they have a higher mPAP (76 mmHg compared to 56.4 mmHg in iPAH patients) [18,56]. As illustrated in Figure 3, one mutation is located on the first cytoplasmic loop, one on the second cytoplasmic loop, three on the first pore domain, six on the second pore domain, and one in the fourth transmembrane domain (Figure 3). 

The consequence of the *KCNK3* mutation for the KCNK3 channel function has been measured by whole-cell patch clamp experiments, in cells overexpressing each different KCNK3-mutated channel (Table 1). As indicated in Table 1, all eight analyzed mutations (T8K, G97R, G106R, E182K, Y192C, G203D, L214R, and V221L) lead to *KCNK3* LOF (Table 1). To date, A114V, K145M, V206L, and A189T have not been evaluated [24,50,54,57]. In addition, the application of ONO-RS-082, which is a phospholipase A2 inhibitor previously shown to activate the KCNK3 channel [58], restores the function of T8K, E182K, and V221L to the same level as the non-mutated channel in basal conditions, while G203D, G106R, and L214R are insensitive to ONO-RS-082 [24,50]. For the V221L mutation, the KCNK3-current is only restored by ONO-RS-082 at extracellular pH 8.3. 

### 1.5. Consequences of KCNK3 Dysfunction for the Physiopathology of PAH

Since the discovery of *KCNK3* mutations in PAH patients, we have found that KCNK3 expression is reduced in lung and pulmonary arteries from iPAH and hPAH patients and that the KCNK3 function is reduced in hPASMCs from iPAH patients. We have also found that KCNK3 expression and function are progressively lost in PASMCs and PAECs isolated from the MCT-PH rat model [30]. Moreover, after the chronic in vivo pharmacological inhibition of KCNK3, we observed an aberrant increase in pulmonary vascular cell proliferation and a significant increase in the right ventricular systolic pressure (RVSP) [30]. In association with the fact that KCNK3 is not functional in mouse PASMCs, Manoury et al. showed that *kcnk3^−/−^* mice do not develop altered PA vasoreactivity [44]. Finally, *kcnk3^−/−^* mice develop similar pulmonary hypertension (PH) after chronic hypoxia exposure to WT mice [59]. Recently, unpublished results have indicated that after chronic exposure to lipopolysaccharide (an inflammatory stressor), *kcnk3^−/−^* mice develop more severe PH than WT mice, which is associated with a more pronounced infiltration of inflammatory cells [60]. 

Using the first *Kcnk3*-LOF-mutant rats generated by CRISPR/Cas9, Lambert et al. demonstrated that *Kcnk3*-LOF-mutant rats develop a spontaneous elevation of RVSP and pulmonary ventricular resistance that is associated with pulmonary vessel neomuscularization. At the molecular level, we found that lungs from *Kcnk3*-LOF-mutant rats are characterized by an increase in proliferative signaling pathways, such as ERK1/2, SRC, and AKT. Hypoxia inducible factor-1α (HIF-1α) is also increased in *Kcnk3*-LOF-mutant rats. These dysregulations are commonly observed in hPASMCs and hPAECs of PAH patients [20]. In addition, after MCT or chronic hypoxia exposure, *Kcnk3*-LOF-mutant rats develop higher PH compared to WT rats. 

Regarding the involvement of Kcnk3 dysfunction in the proliferative phenotype of PAH PASMC, we found that the knockdown of *KCNK3* in control hPASMCs enhances hPASMC proliferation in correlation with an increase of HIF-1α expression and over-phosphorylation of ERK1/2, which are hallmarks of proliferation/apoptosis imbalance and PAH development. Finally, tetramethylrhodamine ethyl ester (TMRE) experiments have indicated that PASMC mitochondria from *Kcnk3*-LOF-mutant rats or siKCNK3 hPASMCs are significantly depolarized and fragmented compared to the control condition [20]. Altogether, these results demonstrate that *Kcnk3*-LOF leads to several defects and dysregulation, thereby increasing the susceptibility to developing PAH. Additionally, using the whole-cell patch-clamp technique, we found that the KCNK3 current is strongly reduced in freshly isolated PASMCs from *Bmpr2*-LOF rats compared to WT PASMCs. In association with this decreased KCNK3 function, we observed a significant depolarization of the Em in PASMCs from *Bmpr2*-LOF rats [61].

Han et al. recently found that resistin-like molecule-β (RELM-β) lung protein expression is strongly increased in chronic hypoxia rats. Using hPASMCs, these authors found that RELM-β promotes hPASMC proliferation by downregulating KCNK3 expression. Indeed, RELM-β overexpression reduces KCNK3 expression and enhances hPASMC proliferation, while siRNA against RELM-β increases KCNK3 expression and reduces hPASMC proliferation [62]. These results suggest that RELM-β is a key player, modulating KCNK3 in PH induced by chronic hypoxia. Previously, Angelini et al. found that RELM-β protein expression is strongly increased in scleroderma-associated PH lungs compared to a control, while RELM-β expression is unchanged in the lungs of iPAH patients, highlighting that RELM-β contributes to the development of scleroderma-associated PH [63]. The contribution of KCNK3 dysfunction in scleroderma-associated PH is unknown; however, we could hypothesize that RELM-β overexpression should reduce KCNK3 expression in the lungs of scleroderma-associated PH patients. Further experiments are needed to investigate the contribution of KCNK3 in scleroderma-associated PH.

## 2. ATP Binding Cassette Subfamily C Member 8 (ABCC8)

*ABCC8* codes for sulfonylurea receptor 1 (SUR1), which is a member of the ATP binding cassette (ABC) family, comprised of a large group of membrane transporters [64]. SUR1 is a regulatory subunit of KATP channels.

KATP channels are inhibited by intracellular ATP (100 µM–10 mM) [65], and this inhibition is mediated by the coupling of Mg^2+^ with ATP [66]. KATP forms a hetero-octameric conformation composed of four inward-rectifier-K^+^ channel subunits (Kir6.x), which represent the pore forming subunits, and four regulatory subunits composed of Sur.x (sulphonylurea receptor) (Figure 3).

There are two types of Kir6.x subunits (Kir6.1 and Kir6.2) and two types of Sur.x subunits (SUR1 and SUR2), and there are two splice variants of SUR2 (SUR2A and SUR2B). The KATP subunit can co-assemble in different manners, depending on the tissue type. On human chromosome 11, *ABCC8* encodes SUR1 and *KCNJ11* encodes Kir6.2. On human chromosome 12, *ABCC9* encodes SUR2 and *KCNJ8* encodes Kir6.1 [67].

### 2.1. SUR1 Properties and Mechanisms of Regulation

**Figure 4:**
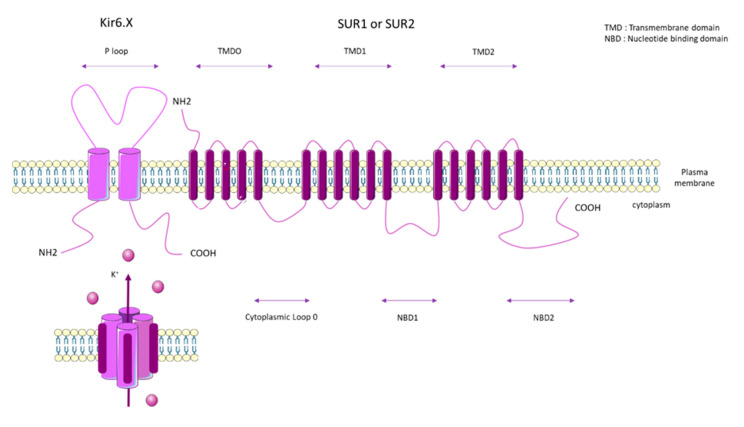
General molecular architecture of ATP-sensitive potassium channels (KATP) channels. The KATP form in a hetero-octameric conformation is composed of four inward-rectifier-K^+^ channel subunits (Kir6.x), which represent the pore forming subunits, and four regulatory subunits (Sur.x, sulfonylurea receptor). There are two types of Kir 6.x subunits (Kir6.1 and Kir6.2) and two types of Sur.x subunits (SUR1 and SUR2), and there are two splice variants of SUR2 (SUR2A and SUR2B). KATP subunits co-assemble in different manners, depending on the tissue type. TMD: Transmembrane domain. NBD: Nucleotide binding domain.

SUR1 is composed of three transmembrane domains (TMD0, TMD1, and TMD2), two nucleotide-binding domains (NBD1 and NBD2), and one cytoplasmic loop (Figure 4). The major role of SUR1 is as a regulator of the Kir6.2 K^+^-channel. SUR1/Kir6.2 is regulated by the metabolic state of the cells as an increase of intracellular ATP leads to channel inhibition. ATP binds to the NBD domains of SUR1 and Kir6.2. Intracellular AMP stimulates the activation of KATP (SUR1/Kir6.2). The AMP/ATP ratio is a key regulator of KATP [68,69].

SUR1 is modulated by the following two classes of drugs: (1) Sulfonylureas, which inhibit KATP opening, that bind to the NBD loop (glibenclamide, tolbutamide, and mitiglinide) [70], and (2) SUR1/Kir6.2, which is activated by K_ATP_ channel openers. SUR1 is highly activated by diazoxide, barely activated by pinacidil, and never activated by nicorandil [71]. Kir6.2 is activated by intra- and extracellular Zn^2+^ [72]. KATP channels are activated by ET-1, which acts as a vasodilator on vascular smooth muscle cells when mediated by H2S (an endothelial-derived factor) [73]. In HEK293 cells, PKC inhibits K_ATP_ [74]. Moreover, in rat mesenteric arterial smooth muscle cells, angiotensin II-mediated PKA and PKC inhibition leads to the closure and inhibition of K_ATP_ [75]. Similarly to KCNK3, KATP channels are known to be activated by PKA- and PKG-dependent phosphorylation [76], suggesting that KATP activation contributes to the vasorelaxating effects of prostanoids and PDE5 inhibitors or guanylate cyclase activators. The regulatory mechanisms of SUR1/Kir6.2 are summarized in Figure 2.

### 2.2. SUR1 Function and Expression in Pulmonary Vasculature and RV

SUR1 and Kir6.2 are mostly co-assembled together, and they are expressed in the brain [77], heart [78], and lung vascular tissues (PAECs and PASMCs) [79,80]. SUR1 also co-assembles and regulates transient receptor melatonin 4 (TRPM4), which is a non-selective cationic channel [81]. However, TRPM4 is expressed in PASMCs, supporting the hypothesis of SUR1/TRPM4 co-assembly in pulmonary vasculature [82]. In cerebral arteries, TRPM4 plays a critical role in the myogenic constriction of cerebral arteries by participating in Em [83]. By regulating the TRPM4 function, we hypothesized that *ABCC8*-LOF also regulates the Em of PASMC and consequently contributes to vasoconstriction and pulmonary artery remodeling. SUR1 can also be associated with Kir6.1 [84,85].

In smooth muscle cells from pig coronary arteries, hypoxia exposure activates K_ATP_ (glibenclamide K^+^-sensitive current) [86]. In femoral smooth muscle cells, K_ATP_ is activated by anoxia, but not by hypoxia [87]. 

SUR1/Kir6.2 are expressed in the human heart, especially in the atria and ventricle [88], including RV tissues [80]. In mice, SUR1/Kir6.2 are mostly involved in atrial myocyte sarcolemmal KATP, whereas SUR2A/Kir6.2 generate ventricular KATP [88]. In humans and in larger animals, SUR1 contributes to sarcolemmal KATP in ventricular and atrial myocytes [89]. The opening of cardiac KATP channels reduces action potential repolarization and the refractory period [88]. 

### 2.3. ABCC8 Mutations in PAH

In 2018, whole-exome sequencing discovered 12 different heterozygous mutations in *ABCC8*, which encodes SUR1, in PAH patients [79]. To date, 11 missense mutations and one splice variant have been identified in PAH patients.

**Figure 5:**
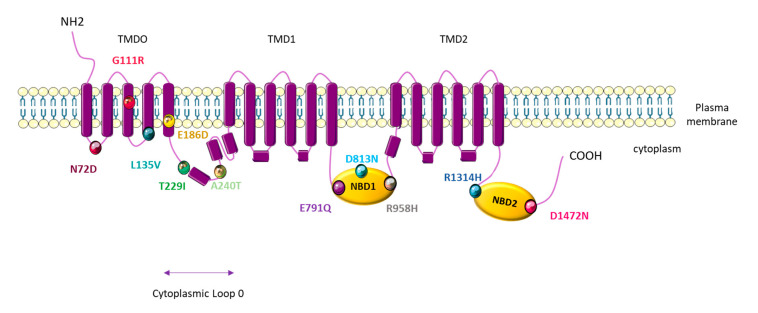
Topological analysis of the human *ATP-binding cassette subfamily C member 8* (ABCC8)/sulfonylurea receptor 1 (SUR1) channel. Positions indicate the mutations identified by Bohnen M et al. [79].

PAH patients that carry the *ABCC8* mutation are younger at diagnosis (14 years compared to 42 years for iPAH patients), but have a similar mPAP to iPAH patients (47.6 mmHg for *ABCC8*-mutated patients and 56.4 mmHg for iPAH patients) [79]. These mutations are localized in amino acid residues well-conserved across species and localized at crucial regions in the protein (five in NBD loops 1 and 2, two in the cytoplasm loop, and four in the TMD0) (Figure 5).

PAH patients with the *ABCC8* mutation generally do not carry other gene mutations, except for one patient, who has been shown to also carry a *TBX4* mutation and develop PAH at an early stage (<1 year). Four rare variants (p.G111R, p.L135V, p.D813N, and p.D1472N) have been described in congenital hyperinsulinism, and two other variants (p.T229I and p.R1314H) have been described in neonatal diabetes mellitus. However, these patients with rare variants did not develop one of the above described pathologies. It is well-known that diabetes induces endothelial dysfunction in systemic vessels and that diabetic patients have a higher risk of developing myocardial infarction and stroke. However, the effect of diabetes on the pulmonary circulation is unclear. Reza Movahed et al. demonstrated that patients with diabetes mellitus have a higher prevalence of pulmonary embolism and pulmonary hypertension [90]. Based on the REVEAL registry, diabetes is found to be a common comorbidity in US patients with PH [91]. Additionally, the long-term survival of diabetic PAH patients is worse than that of non-diabetic PAH patients [92]. 

By patch-clamp recordings and Rubidium (^86^Rb^+^) efflux assays in cells overexpressing each different mutated-SUR1 + WT-Kir6.2, Bohnen et al. found that 11 out of 12 mutations led to a severe decrease in the SUR1/Kir6.2 current [79]. Through ^86^Rb^+^ assays in basal metabolic and inhibited metabolic conditions, these researchers measured a significant decrease in SUR1/Kir6.2 activity for three SUR1 mutants in basal conditions (A240T, D813N, and D1472N) and a significant decrease in SUR1/Kir6.2 activity in inhibited metabolic conditions for five mutations (L135V, A240T, D813N, R958H, and D1472N). Altogether (patch-clamp and Rb assays), these results indicate a significant decrease in the basal or maximal channel activity for all SUR1 mutants tested. Importantly, the function of L135V, E186D, A240T, E791Q, D813N, R958H, R1314H, and D1472N is restored in the presence of diazoxide (100 µM) (Table 2) [79].

Mutations in *ABCC8* and *KCNJ11* are the major cause of neonatal diabetes mellitus [93] and congenital hyperinsulinism [94]. These pathologies are generally treated with sulfonylurea therapy, which leads to a better regulation of insulin secretion and control of glycemia. 

### 2.4. Consequences of ABCC8 Dysfunction for the Physiopathology of PAH 

Currently, there are only a few studies on KATP channels in pulmonary circulation and their implication in PH.

*kcnj11*-deficient mice were generated to study the implication of the KATP channel in pancreatic β-cells. Surprisingly, these mice do not develop the expected hyperglycemic phenotype [95]. *kcnj11^−/−^* mice do not have any obvious cardiovascular defects, but are more sensitive to exercise and stress [96].

*kcnj11^−/−^* mice submitted to left ventricular (LV) pressure overload induced by transverse aortic constriction (TAC) present an abnormal prolongation of the LV cardiomyocyte action potential and an increase in the LV diastolic pressure compared to WT TAC [97]. These results demonstrate a protective role of the KATP channel in hypertensive conditions. In another experimental model of hypertension induced by unilateral nephrectomy and mineralocorticoid salt challenge to mimic chronic hyperaldosteronism, *kcnj11^−/−^* mice exhibit a reduced survival rate associated with a more rapid development of cardiac remodeling and heart failure compared to WT mice [98]. 

*abcc8^−/−^* mice were generated to determine the role of KATP in insulin secretion. However, *abcc8^−/−^* mice do not have dysregulated insulin secretion [99,100]. 

Recently, our preliminary results indicated that SUR1 and Kir6.2 expression levels are maintained in the lungs of iPAH and hPAH patients and three different experimental PH rat models. Myograph experiments on isolated pulmonary arteries (PAs) from non-PAH patients and control rats suggest that SUR1/Kir6.2 are implicated in PA tone regulation as SUR1 activation induces PA relaxation and SUR1 inhibition predisposes to PA vasoconstriction. We also found that *ABCC8* mRNA expression is decreased in the RV from PAH patients and MCT-PH rats [80], but that *KCNJ11* mRNA expression is unchanged in the RV from PAH patients. In addition, *Kcnj11* mRNA expression is decreased in rat hearts and in H9c2 cells under hypoxia conditions [101].The reduction of ABCC8 expression in the RV from PAH patients and MCT-PH rats may contribute to abnormal PH-RV cardiomyocyte excitability, contractility, and metabolism [43].

## 3. Other Genetic Alterations in Genes Coding for K^+^ Channels

### 3.1. ATP Binding Cassette Subfamily C Member 9 (ABCC9)

SUR2, encoded by *ABCC9*, can be spliced into the following two isoforms: SUR2A or SUR2B. SUR2A is mostly co-assembled with Kir6.2 and is expressed in cardiac tissues and skeletal muscles [102]. SUR2B is more often co-assembled with Kir6.1 and is expressed in several tissues, including vascular smooth muscle cells [69]. SUR2B/Kir6.1 are expressed in hPASMCs [103], where they are inhibited by PKC [104].

SUR2B/Kir6.1 are also expressed in endothelial cells from the bovine aorta, guinea pig coronary artery, and human coronary artery [105,106].

SUR2A/Kir6.1 are mostly expressed in the heart, where they are well-known to be cardioprotective [107].

*ABCC9* mutations are the major genetic cause of Cantu Syndrome. Cantu Syndrome is characterized by congenital hypertrichosis, osteochondroplasia, and cardiac defects, such as cardiomegaly, dilated vasculature, tortuous vasculature, and pericardial effusion. Interestingly, some patients have also developed PH [108,109]. To date, 12 different mutations in *ABCC9* have been identified in Cantu Syndrome. Electrophysiological recordings have demonstrated that *ABCC9* mutations are gain-of-function mutations [108,109,110,111,112]. The primary opening of SUR2A/Kir6.1 leads to systemic vasorelaxation and hypotension, and the secondary opening of SUR2A/Kir6.1 leads to compensatory cardiac hypertrophy and hypercontractility [113].

The development of PH observed in Cantu Syndrome patients may be attributed to these cardiovascular disorders. Some patients who have developed LV hypertrophy may also be diagnosed with PAH due to LV disease [67].

A potential treatment for Cantu Syndrome patients with PH may be sulfonylurea receptor inhibitors, such as glibenclamide or mimoxidil. However precaution should be taken due to the potential side effect on SUR1 in the pancreas [108,109] and lung [79].

### 3.2. KCNA5 (Voltage-Gated K^+^ Channels 1.5: Kv1.5)

*KCNA5* encodes Kv1.5, and Kv channels are involved in the control of cell excitability contributing to the regulation of Em. Kv channels are activated by membrane depolarization, and their opening regulates [Ca^2+^]_i_ through the inhibition of voltage-gated Ca^2+^ channels [114].

**Figure 6:**
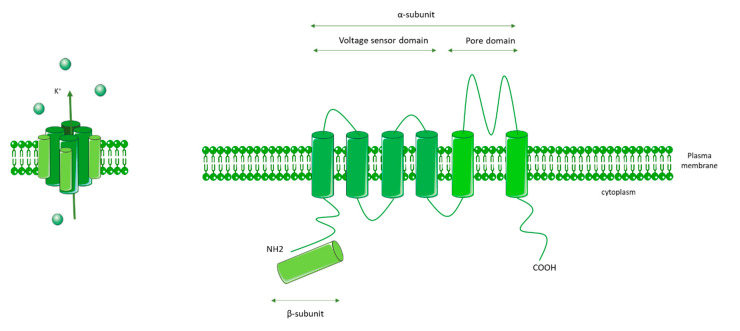
General molecular architecture of voltage-gated K^+^ channels (Kv channels). Kv channels are comprised of a voltage sensor domain (VSD), which detects changes in the transmembrane voltage, and a pore domain (PD). The VSD and PD are comprised of six transmembrane domains. Kv channels are also comprised of a β subunit. Kv channels form as homo- or heterotetramers composed of four α subunits and four β subunits.

Kv channels are formed by an α pore subunit (Kv1–Kv12) composed of a voltage sensor domain (VSD) that detects changes in the transmembrane voltage, as well as a pore domain (PD). VSD and PD are comprised of six transmembrane domains. Kv channels also contain a β subunit, and they form as homo- or heterotetramers comprised of four α subunits and four β subunits (Figure 6).

The putative role of *KCNA5* single nucleotide polymorphisms (SNPs) in PAH has been investigated [115,116]. Indeed, Remillard et al. have identified 17 SNPs in iPAH patients and 12 SNPs have been identified in the 5′ untranslated region (UTR), 2 SNPs in the translated region of *KCNA5*, 2 SNPs in the 3′ UTR region, and 1 SNP in downstream *KCNA5.* Interestingly, the frequency of these two last SNPs in PAH patients with anorexigen history was significantly increased compared to in PAH patients without anorexigen history. These SNPs are localized in the promoter and translated regions of *KCNA5*, suggesting that they should alter the expression of Kv1.5 [116].

Four additional SNPs were identified in a cohort of PAH patients with systemic sclerosis. In this systemic sclerosis cohort, the *KCNA5* rs10744676 variant was associated with protection against PAH [115]. The bioinformatics predictions suggest that this SNP should influence the transcription of *KCNA5*.

However, the functional consequences of all of these identified SNPs in *KCNA5* have not yet been addressed.

In opposition, a meta-analysis study concluded that there is no relationship between SNPs in the *KCNA5* gene and the development of PAH [117].

Kv1.5 is expressed in smooth muscle cells of several tissues and in hPASMCs. A hypoxic environment downregulates Kv1.5 in PASMCs, which is associated with membrane depolarization and an increase of intracellular Ca^2+^ influx [15,118,119]. In response to chronic hypoxia, HIF1-α, which is the master transcription factor activated by hypoxia, mediates the downregulation of Kv1.5 expression. Bonnet et al. found, in spontaneous PH rats (fawn-hooded rats), that HIF1-α is overexpressed and the Kv1.5 expression/function are reduced. After the overexpression of a dominant negative HIF1-α, they found that Kv1.5 expression/function were restored, demonstrating the implication of HIF1-α in the downregulation of Kv1.5. In the same study, they also demonstrated that HIF1-α was stabilized due to a pseudo-hypoxic state mediated by mitochondrial dysfunctions [120,121].

The reduced expression of Kv1.5 using antisense oligonucleotides or siRNA in PASMCs causes Em depolarization and increases the cytosolic Ca^2+^ concentration. The pharmacological inhibition of Kv channels by 4-Aminopurydine (4-AP) and correloide induces Em depolarization and stimulates PAMSC contraction [119,122,123]. Moreover, the PASMCs isolated from *kcna5*^−/−^ mice are depolarized compared to PASMCs isolated from WT mice [124]. Consequently, the hypoxic pulmonary vasoconstriction in the lungs and pulmonary arteries of *kcna5*^−/−^ mice is reduced compared to that in WT mice [124]. Moreover, Archer et al., by using a specific antibody against Kv1.5 in PASMCs, showed that anti-Kv1.5 rapidly inhibited the outward-K^+^ current and rapidly depolarized PASMCs [125].

Interestingly, in PASMCs overexpressing Kv1.5, acute hypoxia reduces outward K^+^ currents and significantly depolarizes the Em potential, demonstrating that the Kv1.5 is a hypoxia-sensitive Kv channel in PASMCs [15,126].

In PAH PASMCs, the decrease in Kv1.5 expression is associated with Em depolarization and an increase in [Ca^2+^]_i_ [122,123,127]. Mutations in *BMPR2* are responsible for hPAH. A decrease in Kv1.5 mRNA expression is found in lungs from *BMPR2*-mutated patients. In hPASMCs treated with BMP2, the Kv1.5 expression and function are increased [128].

Conversely, the overexpression of hKv1.5 in rat PASMCs causes membrane hyperpolarization, enhancing apoptosis [129].

As mentioned earlier, the pathobiology of PAH includes an exaggerated expression of 5-HT, ET-1, and TXA2 [6,7]. As previously reviewed by Boucherat et al., numerous studies have demonstrated that Kv1.5 could be negatively regulated by the activation of phospholipase C (PLC) through the Gq family G-proteins, which include 5-HT, ET-1, and TXA2. In PASMCs, 5-HT signaling negatively regulates the trafficking and surface expression of Kv1.5 [120,130]. In PASMC, TXA2-induced Kv1.5 inhibition is partly mediated by the PKCζ [120]. Since PLC inhibitor or PKC inhibitor application prevents the effects of 5-HT on the Kv1.5 current, ET-1 was also found to inhibit Kv1.5 channel currents [118]. Considering the contribution of Kv1.5 in Em of PASMCs, Kv1.5 inhibition induces the depolarization of PASMCs, enhancing pulmonary artery vasoconstriction, proliferation, and remodeling [119,120,122,123,130]. Moreover, some reports have demonstrated that serotonin-mediated PKC activation results in Kv1.5 inhibition [119,120]. Kv1.5 may also be regulated by endocytosis mediated by Src kinase [130]. The regulatory mechanisms of Kv1.5 are summarized in Figure 2.

Reduced Kv1.5 expressions and functions are observed in iPAH patients and in several experimental PH models [119,120,122,123,130]. Kv1.5 is also expressed in rat PAECs [131].

The Kv1.5 channel is expressed in human ventricular and atrium tissues [132,133]. In human and rat atria, Kv1.5 mediates an ultrarapid outward K^+^-current (IKur current), which contributes to atrial repolarization [133]. Consistent with the key role of Kv1.5 in the human atrium, the *KCNA5*-LOF mutation causes atrial fibrillation [134]. In ventricular myocytes from mice, Kv1.5 contributes to IKslow [135].

## 4. Potential Therapeutic Targets

### 4.1. KCNK3

As the loss of the KCNK3 function is a characteristic of iPAH and hPAH, KCNK3 may be a potential therapeutic target for treating PAH. We found that the in vivo long-term activation of KCNK3 by ONO-RS-082 (50 mg/kg/day; preventive treatment, day 0 to day 21) reduces the development of PH in the MCT-PH model. In contrast, in vivo short-term KCNK3 activation by ONO-RS-082 (curative treatment) fails to reduce PH symptoms, which is attributed to the complete loss of KCNK3 expression in MCT-PH rats at days 14 to 21. These findings reveal that in vivo KCNK3 activation may be effective at ameliorating PH when KCNK3 is expressed in Pas [30], suggesting that the pharmacological activation of KCNK3 may help treat PAH patients who have residual KCNK3 expression. Because the ONO-RS-082 compound is also a phospholipase A2 inhibitor, however, the use of ONO-RS-082 in humans may have deleterious side effects, further indicating the need for the development of more specific activators of KCNK3. Restoring KCNK3 pulmonary vascular expression by gene therapy may be an alternative strategy. In this way, and as suggested by Bohnen et al., forcing the expression of KCNK9 in pulmonary vasculature may be an additional strategy because the overexpression of KCNK9 may restore the function of *KCNK3*-mutated channels by co-assembly [24].

### 4.2. KATP

The major treatment for congenital hyperinsulinism and neonatal diabetes patients carrying *ABCC8* or *KCNJ11* mutations is diazoxide (SUR1 activator) [136]. Diazoxide is one of the U.S. Food and Drug Administration-approved drugs for treating hyperinsulinemic hypoglycemia [137] and may be an interesting option for PAH treatment. Older findings have demonstrated that the acute activation of SUR1 by diazoxide strongly reduces mPAP in some PAH patients [138,139]. Moreover, our preliminary results demonstrated that the in vivo pharmacological activation of SUR1 by diazoxide attenuates the development of PH in MCT-PH rats [80]. However, several reports have indicated that hyperinsulinemic hypoglycemic infants treated with diazoxide develop PH [140,141,142,143,144]. Despite these limitations, our preliminary results suggest that the pharmacological activation of SUR1 should be considered in PAH, indicating the need for more specific molecules for SUR1 activation in humans.

An alternative strategy may be to globally target KATP, as suggested by Zhu et al., who reported that treatment with iptakalim, a new KATP opener (SUR2/Kir6.1), attenuates PH induced by CH exposure [145]. Iptakalim reduces endothelin-induced proliferation in PASMCs [146] and endothelial dysfunction in rats [147]. Together, these results indicate that targeting KATP (SUR1 or SUR2) channels in PAH may also be a promising strategy for treating PAH.

### 4.3. Kv1.5

Because the Kv1.5 expression and function in pulmonary vasculature in PAH patients are severely impaired, an interesting strategy may be gene therapy. Pozeg et al. demonstrated that the in vivo gene transfer of *KCNA5* attenuates PH induced by chronic hypoxia exposure in rats [148].

## 5. Conclusions

PAH remains an incurable disease, representing a major human and social burden. The identification of genetic variations in PAH helps the scientific community to better understand the pathophysiology of PAH and aids in the identification of new potential therapeutic targets. With the evolution of genome sequencing technology, LOF mutations have been identified in *KCNK3* and in *ABCC8* in the last 7 years, suggesting that these molecules play a key role in the development of hPAH and iPAH, which highlights their therapeutic potential for PAH. Regarding the downregulation of K^+^ channels in PAH, novel screening approaches, such as testing drugs, to restore the expression and function of KCNK3, SUR1, or Kv1.5 channels should be considered. Because KCNK3, SUR1, and Kv1.5 are ubiquitously expressed in human tissues, however, a specific pharmacological therapy targeting each of them should be carefully considered in preclinical development to avoid side effects.

## Figures and Tables

**Table 1:** Potassium channel subfamily K member 3 (KCNK3) mutations identified in PAH patients and their consequences for the KCNK3/TASK-1 channel function.

KCNK3 Mutation (AA)	KCNK3 Mutation (Nucleic Acid)	Number of PAH Patients Carrying the Mutation	Number of Healthy Carrier	Zygosity	Function (Patch Clamp)	Function Restored by ONO-RS-082	References
T8K		1		Heterozygous	loss	yes	[50]
G97R	289 G > A	2	1	Heterozygous	loss	/	[50]
G106R	316 G > C	2	1	Heterozygous and homozygous	loss	no	[51]
A114V	341C > T	1		Heterozygous	/	/	[53]
K145M	434 A > T	1		Heterozygous	/	/	[52]
E182K		1		Heterozygous	loss	yes	[50]
A189T	565 G > A	1		Heterozygous	/	/	[52]
Y192C		1		Heterozygous	loss	/	[50]
G203D	608 G > A	6	1	Heterozygous	loss	no	[50,55]
V206L	616 G > T	1		Heterozygous	/	/	[54]
L214R	641 T > G	1		Heterozygous	loss	no	[51]
V221L	661 G > C	1		Heterozygous	loss	/	[50]

**Table 2:** ABCC8 mutations identified in PAH patients and their consequences for the Sur1/Kir6.2 channel function.

*ABCC8* Mutation (AA)	*ABCC8* Mutation (Nucleic Acid)	Number of PAH Patients Carrying the Mutation	Zygosity	Function		Function Restored by Diazoxide
				(Patch Clamp)	(Rubidium (86Rb^+^) Efflux Assays)	
N72D	214 A > G	1	Heterozygous	/	/	/
G111R	331 G > A	1	Heterozygous	/	/	/
L135V	403 C > G	1	Heterozygous	loss	decrease	yes
E186D	558 G > T	1	Heterozygous	loss	not decrease	yes
T229I	686 C > T	1	Heterozygous	/	/	/
A240T	718 G > A	1	Heterozygous	loss	decrease	yes
E791Q	2371 G > C	1	Heterozygous	loss	small decrease	yes
D813N	2437 G > A	1	Heterozygous	loss	decrease	yes
R958H	2873 G > A	1	Heterozygous	not loss	decrease	yes
R1314H	3941 G > A	1	Heterozygous	loss	small decrease	yes
D1472N	4414 G > A	1	Heterozygous	loss	decrease	yes
/	2694 T > 2G	1	Heterozygous	/	/	/
